# Application of rapid Nanopore metagenomic cell-free DNA sequencing to diagnose bloodstream infections: a prospective observational study

**DOI:** 10.1128/spectrum.03295-24

**Published:** 2025-03-26

**Authors:** Morten Eneberg Nielsen, Kirstine Kobberøe Søgaard, Søren Michael Karst, Anne Lund Krarup, Mads Albertsen, Hans Linde Nielsen

**Affiliations:** 1 Department of Chemistry and Bioscience, Center for Microbial Communities, Aalborg University, Aalborg, Denmark; 2 Department of Clinical Microbiology, Aalborg University Hospital, Aalborg, Denmark; 3 Department of Clinical Medicine, Aalborg University, Aalborg, Denmark; 4 Department of Emergency Medicine, Aalborg University Hospital, Aalborg, Denmark; Assistance Publique-Hopitaux de Paris Universite Paris Saclay, Clamart, France

**Keywords:** bloodstream infections, metagenomics, cfDNA, Nanopore, bacteremia

## Abstract

Bloodstream infections are a major cause of mortality, often leading to sepsis or septic shock. Rapid initiation of effective antimicrobial therapy is essential for survival; however, the current gold standard for identifying pathogens in bloodstream infections, blood culturing, has limitations with long turnaround time and poor sensitivity. This delay in refining empirical broad-spectrum antimicrobial treatments contributes to increased mortality and the development of antimicrobial resistance. In this study, we developed a metagenomic next-generation sequencing assay utilizing the Oxford Nanopore Technologies platform to sequence microbial cell-free DNA from blood plasma. We demonstrated proof of concept in a prospective observational clinical study including patients (*n* = 40) admitted to the emergency ward on suspicion of bloodstream infection. Study samples were drawn from the same venipuncture as a blood culture sample from the included patients. Nanopore metagenomic sequencing confirmed all microbiological findings in patients with positive blood cultures (*n* = 11) and identified pathogens relevant to the acute infection in an additional 11 patients with negative blood cultures. This proof-of-concept study demonstrates that culture-independent Nanopore metagenomic sequencing directly on blood plasma could be a feasible supplementary test for infection diagnostics in patients admitted with severe infections or sepsis. These findings support further studies on Nanopore metagenomic sequencing for sepsis diagnostics in larger cohorts to validate and expand the results from this study.

**IMPORTANCE:**

This study demonstrates the potential of Nanopore metagenomic sequencing as a rapid, culture-independent diagnostic tool for bloodstream infections, identifying pathogens missed by conventional blood cultures. The study highlights the method’s promise in improving pathogen detection and warrants further validation in larger clinical studies.

## INTRODUCTION

Bloodstream infections (BSIs) are frequent and associated with high mortality due to the development of sepsis (1). The Global Burden of Disease study found that there were 50 million sepsis cases and 11 million sepsis-related deaths in 2017, constituting nearly 20% of all global deaths (2). To treat sepsis, the current international guideline recommends that broad-spectrum empiric antimicrobial treatment should be administered ideally within 1 hour from its recognition (3). However, in 20% of cases, the empiric antimicrobial treatment does not cover the causative pathogen, leading to higher mortality (4, 5). To facilitate targeted and effective antimicrobial treatment to improve sepsis survival, an accurate and rapid identification of the causative pathogen and its antimicrobial susceptibility is crucial. However, gold-standard blood culturing fails to diagnose over 50% of patients with clinical sepsis, possibly due to prior antimicrobial treatment or a microbial load below the limit of detection (6, 7). Furthermore, blood culturing has a turnaround time to pathogen identification of approximately 2 days, which contrasts the urgency to provide effective antimicrobial treatment (8). In addition to the urgency on a patient level, there is an increased focus on improving diagnostics to facilitate effective antimicrobial stewardship programs (9, 10).

This has prompted the development of novel approaches to BSI diagnostics, including multiplex PCR assays, molecular target detection with magnetic resonance, and metagenomic next-generation sequencing (mNGS) of microbial cell-free DNA (cfDNA) in plasma (11–14). Of these, mNGS has gained particular attention due to its high sensitivity and hypothesis-free approach. While cfDNA mNGS has an increased number of relevant detections of pathogens compared to the standard blood culturing, the method has generally been applied on DNA sequencing platforms with long turnaround times that compromise the urgency of diagnosing BSIs.

We leveraged the real-time Nanopore sequencing platform to develop a plasma mNGS assay with a short turnaround time and evaluated its performance in a prospective observational study in a hospital emergency ward setting against gold-standard blood culturing.

## MATERIALS AND METHODS

### Study design

We performed a cross-sectional study in the emergency ward at Aalborg University Hospital, which serves as the reference hospital in the North Denmark Region (catchment population approximately 600,000 inhabitants). Patients were prospectively enrolled upon suspicion of BSI between 1 June and 31 December 2022. For each patient, a blood sample (9 mL K2EDTA) for mNGS was drawn from the same venipuncture as a blood culture for routine diagnostics. Following inclusion, patient metadata was collected from their electronic medical record, which included medical history and diagnosis, clinical microbiology results, biochemical blood parameters, and antimicrobial therapy. As a negative reference group, healthy blood donor samples were collected from the blood bank at Aalborg University Hospital from 12 donors and handled in parallel with the clinical samples.

### Study population

Study inclusion criteria comprised that the patient was admitted to the emergency ward at Aalborg University Hospital upon suspicion of BSI as adjudicated by a requested blood culture. Patients were required to be alert, oriented, and able to read and understand participant information. Patients were excluded from the study if they were younger than 18 years old or were unable to provide written informed consent. Enrollment took place on selected weekdays from 8 a.m. to 4 p.m.

### Patient subgrouping

**Fig 1:**
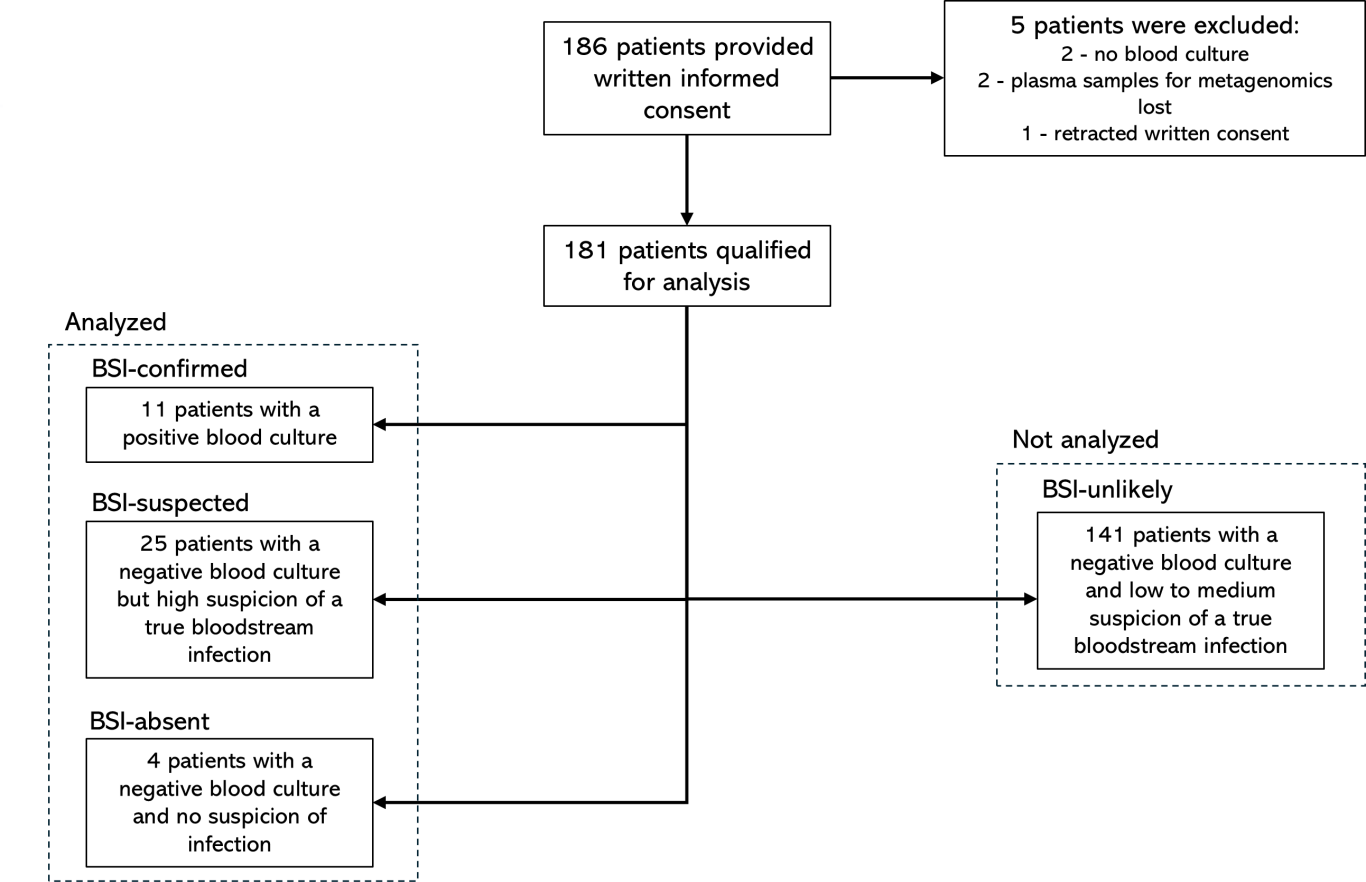
Flowchart of patient inclusion and selection for analysis.

Based on the medical records, patients were subdivided into the following groups as adjudicated by a clinical microbiologist (K.K.S.): patients with a positive blood culture (BSI-confirmed); patients with a negative blood culture but a high suspicion of BSI (BSI-suspected); patients with a negative blood culture and no suspicion of an infectious disease (BSI-absent); and patients with a negative blood culture and low suspicion of a true BSI (BSI-unlikely). Patients were assigned based on all available microbiology test results (blood culture results, urine culture, respiratory samples, etc.) combined with a clinical evaluation of the patients including vital signs (e.g., temperature, pulse, blood pressure, respiration rate), biochemical parameters (e.g., CRP [C-reactive protein], white blood cell count), and imaging (e.g., chest X-ray, CT abdomen and chest, endoscopies). Patients in groups BSI-confirmed, BSI-suspected, and BSI-absent were selected for analysis. Patients in the BSI-unlikely group were excluded from metagenomic sequencing analysis due to limitations in funding. Patients in the BSI-absent group were analyzed to evaluate specificity (Fig. 1).

### Clinical microbiology

All clinical microbiology results were subsequently obtained from patient medical records (wwBakt, Autonik AB, Sweden), and no follow-up analyses were conducted. Clinical microbiology analyses were conducted at the Department of Clinical Microbiology at Aalborg University Hospital. A standard blood culture using two BD BACTEC Plus Aerobic medium and one BD BACTEC Lytic Anaerobic medium glass culture vial were obtained from a peripheral site upon submission and incubated in the BACTEC FX Top instrument (Becton Dickinson AB, Stockholm, Sweden). Species identification was performed using conventional biochemical tests and matrix-assisted laser desorption ionization–time of flight (MALDI Biotyper 3.1, Bruker Daltonics Microflex LT, MBT 6903 MSP Library), with scores ≥2 for identification at the species level. However, *Escherichia coli* was identified based on the growth of red colonies on the CHROMagar Orientation medium and a positive indole test, according to the manufacturer’s instructions.

### Plasma preparation and DNA extraction

Plasma was prepared from study blood samples by centrifugation at 1,600 *g* at 4°C for 10 minutes within 1 hour from blood draw at the Department of Biochemistry at Aalborg University Hospital. The plasma supernatant was subsequently centrifuged again at 16,000 *g* at 4°C for 10 minutes and the new supernatant was obtained and stored at −80°C until analysis. At analysis, plasma samples were thawed, and single-stranded oligonucleotides with lengths from 50 to 200 bp and a distinct, conserved region for bioinformatic discrimination were added in a series of relevant concentrations to allow for absolute quantification of microbial DNA. DNA was extracted from 2 mL plasma using the QIAmp MinElute ccfDNA kit (Qiagen) standard protocol with DNA elution in 30 µL. At least one process control was included in each batch, consisting of 2 mL nuclease-free water (Qiagen). DNA was quantified with the Qubit dsDNA HS Assay Kit on a Qubit 4 Fluorometer (Thermo Fisher). DNA length was assessed with the D1000 High Sensitivity ScreenTape Assay on a TapeStation 4150 (Agilent).

### Library preparation and sequencing

**Fig 2:**
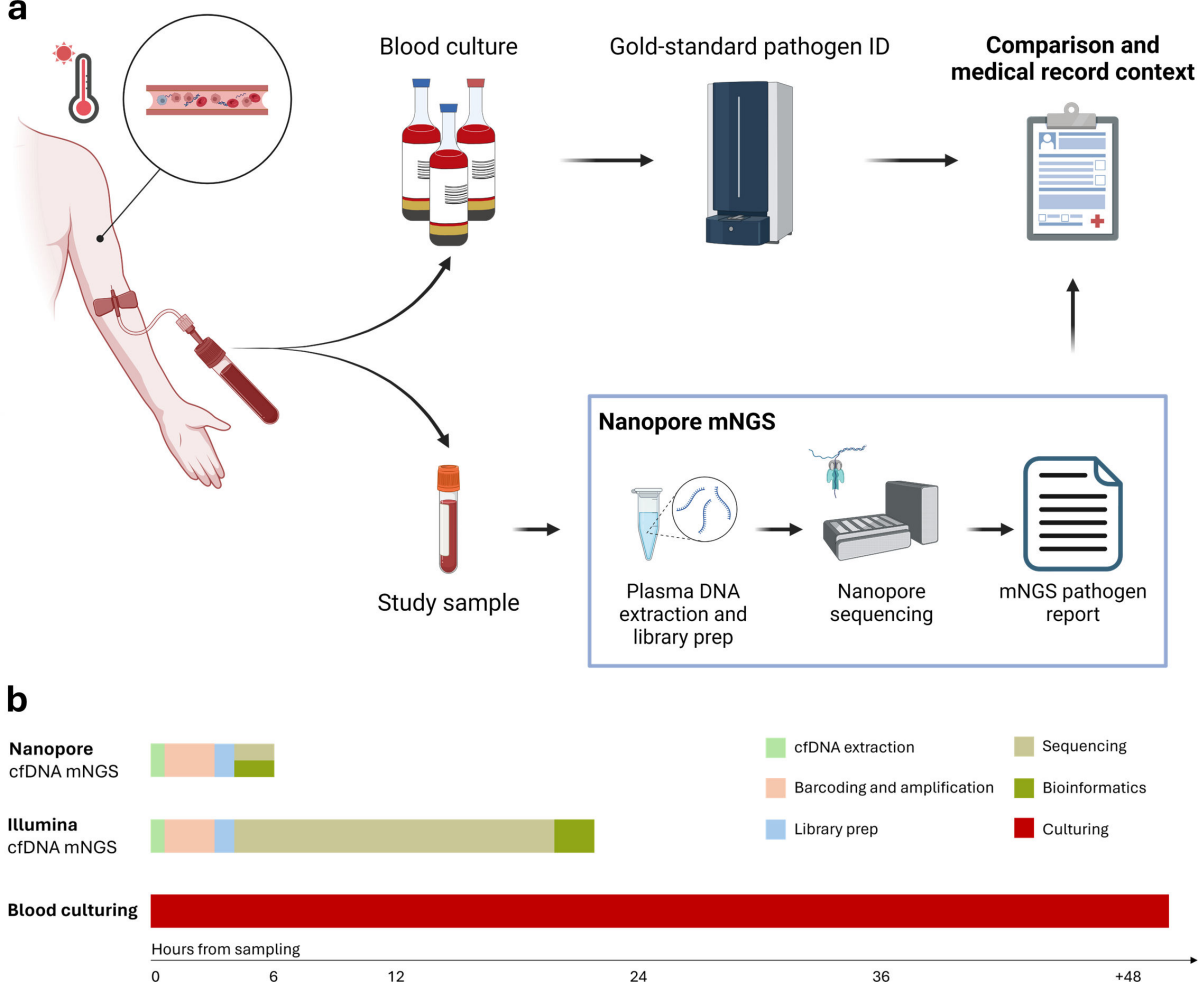
(a) Flowchart of the sample handling workflow for blood cultures and mNGS samples (created with biorender.com). (b) A timeline for BSI diagnostics with Nanopore mNGS (15), Illumina mNGS (14), and blood culturing (8).

Ten to 18 µL of extracted DNA was used as input to the SRSLY DNA NGS Library Preparation PicoPlus Kit (Claret Biosciences). The library preparation was carried out according to the SRSLY protocol for extreme short fragment retention. At least one process control was included in each batch, consisting of 18 µL nuclease-free water (Qiagen). Subsequently, samples were prepared for Oxford Nanopore sequencing with the ligation kit (Oxford Nanopore Technologies, SQK-LSK114), where four or five samples were multiplexed with a total of 200 fmol DNA as input. Libraries were sequenced on a PromethION 24 on R10.4.1 flow cells and basecalled with the super-accurate model (Guppy 7.0.9) with a quality cut-off at a Phred score of 10 applied (Fig. 2).

### Data analysis and bioinformatics

Basecalled reads were demultiplexed, trimmed for adapters, and checked for any remaining barcodes or adapters with Cutadapt (version 4.4) (16). Reads were aligned to the human genome (consecutive rounds of alignment with minimap2 version 2.26, bwa version 0.7.17, and BLAST version 2.15.0). Reads with no significant alignments were aligned with Bowtie2 (-k 10 --mp 2,2 --score-min G,20,8) to a database consisting of Genome Taxonomy Database representative bacterial and archaeal genomes from release 207 and virus (complete genomes) and fungi (all genomes) from NCBI RefSeq release 215 as well as the reference NCBI Homo sapiens genome (T2T) (17–20). All microbial reads were re-evaluated in a last-common-ancestor approach and only accepted if more than 50% of near-best alignments for a read (edit distance <10% of read length and aligned length >90% read length) matched the same genus. Species with a non-uniform genome coverage of reads were discarded. Reads from the single-stranded spike-ins were used to quantify DNA for all identified pathogens in genome equivalents per microliter (GPM). The absolute load of microbial DNA in patient samples was compared to the 12 blood donor samples for each species and included only when the absolute load in a patient sample was five times that observed in the control sample with the highest load of that species.

### Assessment of clinical relevance of mNGS results

To evaluate the relevance of mNGS findings, results were contextualized with the patient’s other microbiological results and overall clinical picture. mNGS results were grouped on a patient level based on the clinical relevance of identifications as adjudicated by two clinical microbiologists (K.K.S. and H.L.N.) (13). “Confirmed” was assigned only when at least one of the pathogens identified by mNGS was also identified by blood culture. “Probable” was assigned when at least one of the pathogens identified by mNGS was also identified in other samples from the suspected infection site or when the mNGS results aligned very well with the clinical picture (e.g., *E. coli* identified in a patient with suspected urinary tract infection [UTI]). “Possible” was assigned when at least one of the pathogens identified by mNGS was consistent with the clinical picture. “Unlikely” was assigned when the pathogens identified by mNGS were considered irrelevant in the context of the acute infection.

### Potential clinical impact analysis

To evaluate the potential impact on patient antimicrobial treatment, the microbial identifications from mNGS were compared to results from routine diagnostics, medical record (suspected site of infection), and the antimicrobials administered prior to and during the relevant admission by two clinical microbiologists (K.K.S. and H.L.N.). As the mNGS analysis was conducted in batches following completed patient inclusion, the impact analysis was based on the turnaround time in a potential real-time setup, which was estimated to be around 6 hours. This aligns with previously reported turnaround times for Nanopore mNGS (15). Patient courses were evaluated for three different potential impact types. Firstly, a change from ineffective to effective antimicrobial treatment was assigned when mNGS results could have led to a correction from ineffective (either due to antimicrobial resistance or the antimicrobial not covering the pathogen) to effective antimicrobial treatment. This was adjudicated only when the first-line antimicrobial against the pathogen identified by mNGS would have been effective. Secondly, an addition of an extra antimicrobial for coverage was assigned when mNGS results could have led to the addition of one or more extra antimicrobials to cover all identified pathogens relevant to the acute infection. Thirdly, extended treatment was assigned when the finding of a pathogen in the blood with DNA sequencing that was already covered by the antimicrobial treatment could have led doctors to consider prolonging IV antimicrobial treatment and admission. Only the patients where pathogens from mNGS were adjudicated as relevant to the acute infection were considered in the impact analysis. In the assessment of impact, all clinical microbiology diagnostics were considered, and e.g., a change to effective treatment was only adjudicated if no relevant diagnostic answers were available at the time (6 hours post admission) to guide the treatment. Negative mNGS answers were not assessed for potential in guiding de-escalations.

### Statistical analysis

Confidence intervals for sensitivity and specificity were calculated using the Clopper-Pearson exact method. If not specified otherwise, a two-sided Wilcoxon-Mann-Whitney test was used to compare distributions. In case of multiple comparisons, the *P*-values were adjusted with the Holm-Bonferroni method. The comparison of mNGS results to gold-standard blood culturing was conducted as follows: a true positive was adjudicated when at least one pathogen identified by mNGS was in accordance with blood culturing; a true negative was adjudicated when no pathogens were identified by either blood culture or mNGS; a false positive was adjudicated when blood culture was negative and mNGS was positive; a false negative was adjudicated when blood culture was positive and mNGS was negative for that pathogen (21). All statistical analyses were conducted in R (version 4.2.0).

## RESULTS

### Patient inclusion and sample selection

During the study period, a total of 186 patients provided written informed consent. Following inclusion, 40 patients were selected for analysis after subdividing the patients into four groups: BSI-confirmed (*n* = 11), BSI-suspected (*n* = 25), BSI-absent (*n* = 4), and BSI-unlikely (*n* = 141), while five patients were excluded (Fig. 1). Only samples from patients in BSI-confirmed, BSI-suspected, and BSI-absent were subjected to mNGS analysis.

### Patient characteristics

A subset of 40 patients (50% female) with a median age of 71 years (interquartile range [IQR]: 59–84) was analyzed using mNGS (Table 1; for detailed metadata, see Table S1). The most common infection types were UTI (*n* = 12) and lower respiratory tract infection (LRTI, *n* = 10). Of the 12 patients admitted with a UTI, 5 were suspected to have pyelonephritis (Table 2). More than half of the 40 patients had one or more comorbidities, including diabetes (*n* = 13), cancer (*n* = 7), and chronic obstructive pulmonary disease (*n* = 5). Emphasizing the success of our BSI categorization, we observed significantly higher CRP levels in the BSI-confirmed (*n* = 11, median: 216 mg/L, *P* = 0.047 and *P* = 0.002, Wilcoxon-Mann-Whitney) and BSI-suspected (*n* = 25, median: 204 mg/L, *P* = 0.035 and *P* < 0.0001, Wilcoxon-Mann-Whitney) groups, compared to BSI-absent (*n* = 4, median: 1.3 mg/L) and BSI-unlikely (*n* = 141, median: 67 mg/L) groups (Fig. S1).

**TABLE 1:** Patient characteristics of the three groups analyzed with metagenomic DNA sequencing.

	Patient subgroups		
	BSI-confirmed (*n* = 11)	BSI-suspected (*n* = 25)	BSI-absent (*n* = 4)
Patient demographics			
Age (years)			
Median (IQR)	72 (68.5–81.5)	67 (59–84)	56.5 (50.5–67.5)
Range	24–92	23–93	46–87
Female, *n* (%)	3 (27)	15 (60)	2 (50)
Comorbidities, *n* (%)			
Any	4 (36)	15 (60)	3 (75)
Diabetes	3 (27)	9 (36)	1 (25)
Cancer	1 (9)	5 (20)	1 (25)
Chronic obstructive pulmonary disease	1 (9)	3 (12)	1 (25)
Alcoholism	1 (9)	1 (4)	1 (25)
Dialysis	0 (0)	0 (0)	0 (0)
Immunosuppressive therapy^*a*^, *n* (%)	0 (0)	2 (8)	0 (0)
Clinical presentation, *n* (%)			
Oral antimicrobials at admission^*b*^	0 (0)	7 (28)	0 (0)
Sepsis according to qSOFA^*c*^ criteria (≥2)	2 (18)	4 (16)	0 (0)
Primary site of infection			
Urinary tract infection	6 (55)	6 (24)	..^*d*^
Lower respiratory tract infection	0 (0)	10 (40)	..
Skin and soft tissue infections	1 (0)	4 (8)	..
Diverticulitis	0 (0)	1 (4)	..
PICC^*e*^e line	0 (0)	1 (4)	..
Idiopathic pancreatitis	1 (9)	0 (0)	..
Unknown	3 (27)	3 (12)	..
30-day all-cause mortality	0 (0)	1 (4)	0 (0)

^*a*^ Immunosuppressive therapy covers ATC codes L04 and H02.

^*b*^ Oral antimicrobials administered at the general practitioner in relation to the relevant infection.

^*c*^ qSOFA, Quick Sequential Organ Failure Assessment (22).

^*d*^ .., Not relevant.

^*e*^ PICC, peripherally inserted central catheter.

**TABLE 2:** Results from conventional microbiological tests and mNGS for the three groups.

Patient group	Patient	Suspected primary site of infection	Blood culture results (positive bottles)	Other positive microbiology results site: species	mNGS result species (GPM)	mNGS result category
BSI-confirmed	p001	UTI	*Enterococcus faecalis* (3/3)	Urine: *E. faecalis*	*E. faecalis* (1)	Confirmed
	p002	UTI	*E. coli* (2/3)	Urine: *E. coli* Urine: *Enterococcus* spp.	*E. coli* (20.8)	Confirmed
	p020	UTI	*E. coli* (2/3)	Urine: *E. coli*	*E. coli* (1.6)	Confirmed
	p028	UTI	*E. coli* (3/3)	Urine: *E. coli*	*E. coli* (41.3)	Confirmed
	p098	Idiopathic pancreatitis	*E. coli* (3/3)	..^*a*^	*E. coli* (0.9)	Confirmed
	p104	Infection with unknown focus	*Staphylococcus epidermidis* (3/3)	..	*S. epidermidis* (52.4) KSHV^*b*^ (1)	Confirmed
	p139	Infection with unknown focus	*E. coli* (3/3)	..	*E. coli* (102.2) *Enterococcus faecium* (0.7) CMV^*c*^ (26.6)	Confirmed
	p141	Knee	*Staphylococcus aureus* (3/3)	Urine: *S. aureus* Joint: *S. aureus*	*S. aureus* (31)	Confirmed
	p172	UTI (pyelonephritis)	*E. coli* (1/3)	Urine: *E. coli*	*E. coli* (59.4)	Confirmed
	p175	Infection with unknown focus	*S. epidermidis* (3/3)	Urine: *Enterococcus spp*.	*S. epidermidis* (53.8)	Confirmed
	p183	UTI	*E. coli* (3/3)	Urine: *E. coli* Feces: *Clostridioides difficile*	*E. coli* (320.8) *Citrobacter koseri* (2.4)	Confirmed
BSI-suspected	p012	Infection with unknown focus	..	Urine: *E. coli*	..	Negative
	p018	PICC-line infection	..	..	..	Negative
	p019	LRTI	..	..	*E. coli* (58.7) *H. influenzae* (13.4) *Rothia dentocariosa* (4.5) *Streptococcus anginosus* (4.6) *Veillonella atypica* (6.5)	Probable
	p022	Infection with unknown focus	..	..	..	Negative
	p027	Skin (erysipelas)	..	..	..	Negative
	p047	LRTI	..	Urine: *Pseudomonas aeruginosa*	..	Negative
	p049	LRTI	..	..	*Proteus mirabilis* (0.5)	Possible
	p072	UTI	..	..	*E. coli* (2.8)	Probable
	p091	UTI (pyelonephritis)	..	Urine: *E. coli*	*E. coli* (1.8)	Probable
	p092	UTI (pyelonephritis)	..	Urine: *E. coli*	*E. coli* (0.9) *Helicobacter pylori* (13.8)	Probable
	p105	LRTI	..	BAL: *P. aeruginosa*	*P. aeruginosa* (121.6)	Probable
	p106	LRTI	..	Sputum: *Streptococcus pneumoniae* Sputum: RS virus^*d*^	..	Negative
	p114	LRTI	..	..	..	Negative
	p127	Diverticulitis	..	..	*Raoultella planticola* (0.6)	Probable
	p128	LRTI	..	..	*S. pneumoniae* (3.5)	Probable
	p136	Psoas	..	Abscess: *E. coli* Abscess: *Candida albicans*	..	Negative
	p140	UTI	..	Urine: *E. coli*	*E. coli* (5.9)	Probable
	p143	Decubitus os sacrum	..	Urine: *Enterococcus* spp. Wound: *Proteus* spp.	*C. difficile* (0.5)	Unlikely
	p146	Skin (erysipelas)	..	Wound: *S. aureus*	..	Negative
	p150	LRTI	..	..	..	Negative
	p151	Infection with unknown focus	..	..	..	Negative
	p156	LRTI	..	Urine: *Klebsiella pneumoniae*	..	Negative
	p162	LRTI	..	Urine: *E. coli* Sputum: *Haemophilus influenzae*	..	Negative
	p164	UTI (pyelonephritis)	..	Urine: *E. coli*	*E. coli* (8)	Probable
	p173	UTI (pyelonephritis)	..	Urine: *Enterococcus* spp.	*Klebsiella* spp.^*e*^ (5.8) *Acinetobacter bereziniae* (1) *Brevibacterium paucivorans* (2) *Leuconostoc pseudomesenteroides* (1.1) *Pantoea dispersa* (1.1) *Serratia* spp.^*e*^ (3.1)	Probable
BSI-absent	p016	..	..	..	..	Negative
	p059	..	..	..	..	Negative
	p068	..	..	..	..	Negative
	p120	..	..	Skin: *S. aureus*	..	Negative

^*a*^ .., Not relevant.

^*b*^ KSHV, Kaposi’s sarcoma-associated herpesvirus.

^*c*^ CMV, cytomegalovirus.

^*d*^ RS virus, human respiratory syncytial virus.

^*e*^ Could not be determined to species level with mNGS.

### Clinical microbiology

The 11 positive blood cultures from patients in the BSI-confirmed group identified *E. coli* (*n* = 7), *Staphylococcus epidermidis* (*n* = 2), *Enterococcus faecalis* (*n* = 1), and *Staphylococcus aureus* (*n* = 1), reflecting that most patients in this group were admitted with urosepsis (Table 2). Of the 25 patients in the BSI-suspected group, 14 (56%) had positive cultures from urine (*n* = 10), sputum (*n* = 2), bronchoalveolar lavage (*n* = 1), or other sites (*n* = 3), while the remaining patients in this group had no positive microbiological tests to guide the antimicrobial treatment. Pathogens identified in other cultures for BSI-suspected patients were *E. coli* (*n* = 7), *Pseudomonas aeruginosa* (*n* = 2), and others (*n* = 9).

### Metagenomic DNA sequencing analysis

**Fig 3:**
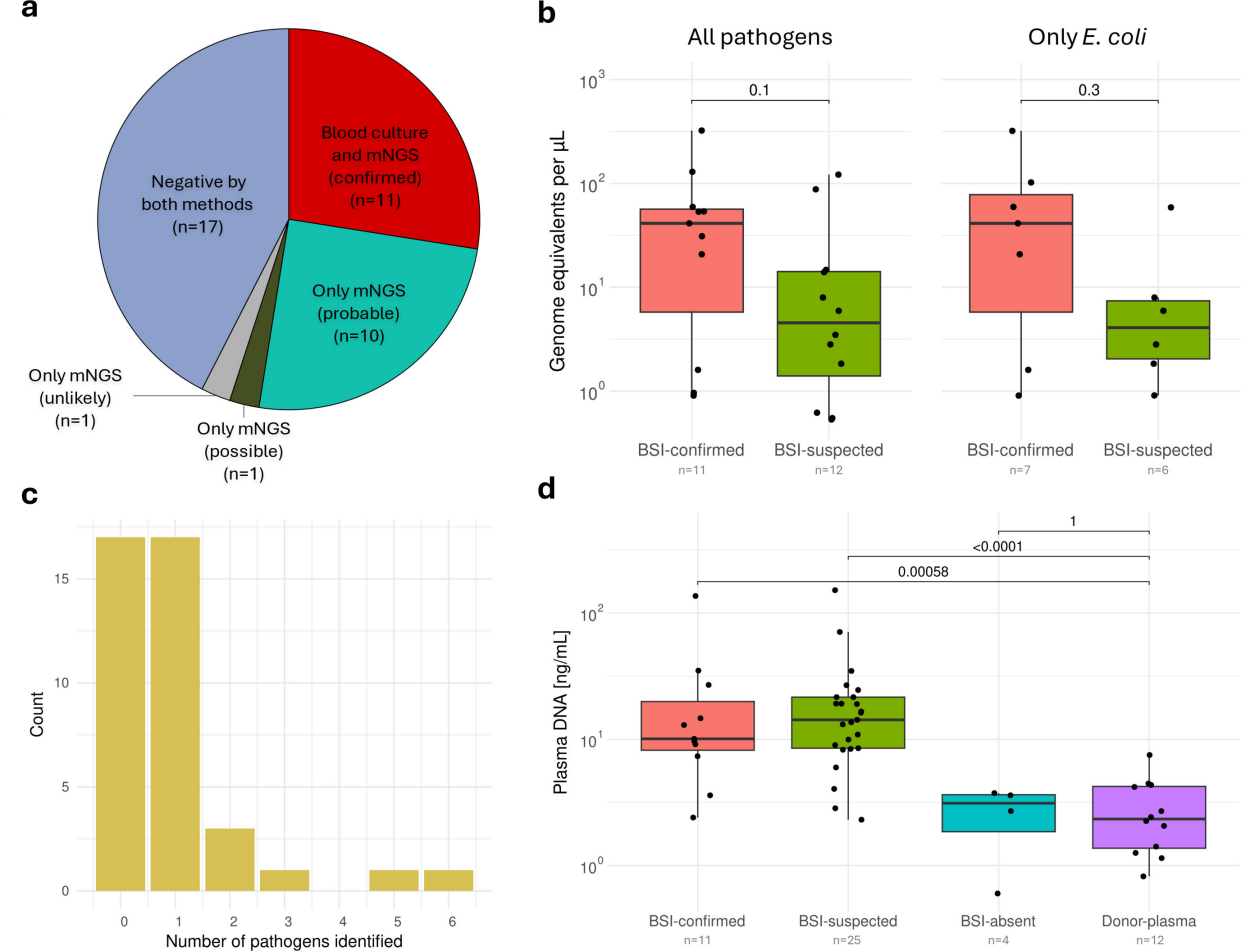
(a) Pie chart summarizing findings from blood culture and mNGS. (b) Total pathogen DNA burden for patients (measured in genome equivalents per microliter) positive by mNGS in BSI-confirmed and BSI-suspected and compared only for *E. coli* where identified. (c) Number of pathogens identified with mNGS per sample. (d) Plasma cfDNA concentrations across the different patient groups and blood donor samples.

From all 40 patients, plasma cfDNA was sequenced (Fig. 2) and patient samples had a mean read depth of 10.1M reads (s.d. 5.6M) with a mean Phred quality of 19.0 (s.d. 0.7) and an average read length N50 of 176 bp (s.d. 11.1 bp) (Table S2). Patients in BSI-confirmed (*n* = 11, median: 10.1 ng/mL) had significantly higher levels of cfDNA in plasma compared to blood donor samples (*n* = 12, median: 2.3 ng/mL) (*P* = 0.00058, Wilcoxon-Mann-Whitney), which was also the case for patients in BSI-suspected (*n* = 25, median: 14.3 ng/mL) (*P* < 0.00001, Wilcoxon-Mann-Whitney) in accordance with previous reports (Fig. 3d) (23).

mNGS confirmed all positive blood culture findings, and three patients in this group had one or two additional pathogens identified (Table 2). The additional findings comprised two bacteria (*Enterococcus faecium* [p139] and *Citrobacter koseri* [p183]) and two viruses (human cytomegalovirus [human betaherpesvirus 5, CMV] [p139] and Kaposi’s sarcoma-associated herpesvirus (human gammaherpesvirus 8, KSHV) [p104]). In the BSI-suspected group, one to six pathogens were identified in 12 of 25 patients (Fig. 3c). Eleven of the 12 patients with a positive mNGS result had one or more pathogens identified that were deemed relevant for the acute infection by a clinical microbiologist and assigned either “Probable” (*n* = 10) or “Possible” (*n* = 1) (Fig. 3a). This included common pathogens like *E. coli*, *P. aeruginosa*, and *Streptococcus pneumoniae*, but also pathogens like *Serratia* spp., *Raoultella planticola*, and *Veillonella atypica*. One patient with a positive mNGS result, interpreted as “Unlikely” to be relevant to the acute infection, had *Clostridioides difficile* identified (p143). Interestingly, in 5 of the 12 patients in the BSI-suspected group with a pathogen identified by mNGS, another microbiological test result supported the finding (Table 2). All patients who received antimicrobials prior to admission (*n* = 7) were in the BSI-suspected group, of which three (p072, p105, and p140) were positive with mNGS (Table S1). No pathogens were identified in the BSI-absent group. Evaluated against blood culture findings, the sensitivity was 100% (confidence interval 71.5%–100.0%) and the specificity was 59% (confidence interval 38.9%–76.5%) at a patient level (Clopper-Pearson exact method). Since most pathogen identifications were deemed relevant to the acute infection, the low specificity is likely due to blood culturing being a poor reference method.

With the single-stranded DNA spike-ins, the load of pathogen DNA in plasma samples was quantified, and pathogens were reported in titers from 0.5 to 321 GPM, which is in accordance with previously reported pathogen DNA titers in BSIs (24). Comparing the load of *E. coli*—the most frequently identified pathogen—across BSI-confirmed (*n* = 7, median: 41.3 GPM) and BSI-suspected (*n* = 6, median: 4.37 GPM), there was no significant difference (*P* = 0.30, Wilcoxon-Mann-Whitney), which was also the case when comparing the total pathogen load in patients from BSI-confirmed (*n* = 11, median: 41.3 GPM) and BSI-suspected (*n* = 12, median: 4.71 GPM) with a positive mNGS test (*P* = 0.10, Wilcoxon-Mann-Whitney) (Fig. 3b).

### Case series

To contextualize the results of mNGS to the patient clinical presentation, we present a more detailed review of selected cases from BSI-confirmed and BSI-suspected groups, who were positive by mNGS. Cases that highlight possibilities and challenges of the method were selected once all results were available. From the BSI-confirmed group, three patients were selected for a more detailed analysis.

The first patient (p001), a 77-year-old male, was admitted with symptoms of septicemia with LRTI as the suspected primary focus and a CRP of 109 mg/L (normal reference range <8 mg/L). The patient was treated with empirical IV cefuroxime in combination with gentamicin. However, the focus was diagnosed after 48 hours as urosepsis with the identification of *E. faecalis* in blood and urine cultures, prompting a change to IV ampicillin. mNGS also identified *E. faecalis* at 1 GPM.

The second patient (p104), a 66-year-old male with Kaposi’s sarcoma and recurrent episodes of spontaneous bacterial peritonitis, secondary to liver cirrhosis, was admitted with acute abdominal pain and an elevated CRP of 41 mg/L, with no obvious signs of infection. Initial treatment included IV piperacillin-tazobactam and oral ciprofloxacin. After 48 hours, blood cultures taken from both a peripheral vein and a peripherally inserted central catheter (PICC) line were positive for *S. epidermidis*, with the PICC line identified as the suspected source. The PICC line had been in place for an extended period, though the exact date of insertion was unknown. This prompted a change in treatment to IV vancomycin, and the PICC line was removed. mNGS identified *S. epidermidis* (52.4 GPM) and KSHV (1 GPM).

The third patient (p139), a 64-year-old male, was admitted with infection with unknown focus and an elevated CRP of 248 mg/L. The patient had a medical history of a renal transplant and received immunosuppressives. *E. coli* was cultured from blood taken at admission after 46 hours, and this was also identified by mNGS at 102 GPM. Additionally, mNGS identified *Enterococcus faecium* (0.7 GPM) and CMV (26.6 GPM). A PET-CT scan identified the infectious focus as multiple infected renal and liver cysts, and the patient was administered IV piperacillin-tazobactam for 31 days with gradual improvement. On day 11 of the hospitalization, a quantitative PCR for CMV was positive with 290 copies/mL.

From the BSI-suspected group, three patients were selected for a more detailed description.

The first patient (p128), a 72-year-old female, was admitted upon suspicion of pneumonia with a CRP of 470 mg/L and a low systolic blood pressure of 99 mmHg. The patient was known with chronic obstructive pulmonary disease and autoimmune hepatitis. The patient was seen by a general practitioner prior to the admission concerning the LRTI, but it was unclear from the medical record if the patient was treated with antimicrobials before admission. All microbiology tests taken at admission (blood culture, urine culture, *S. pneumoniae* urine antigen test) were negative, while *S. pneumoniae* was identified by mNGS at 3.5 GPM. The patient was administered IV benzylpenicillin at admission with a good clinical response.

The second patient (p173) was a 66-year-old female with a medical history of nephrolithiasis treated with double-J stent and numerous episodes of UTIs. She was admitted on suspicion of pyelonephritis presenting a CRP of 164 mg/L. Prior to admission, the patient was treated with ciprofloxacin, and a urine culture obtained 5 days before was positive for *Enterococcus* spp. (1 × 10e4 CFU/mL). However, the blood and urine cultures taken at admission were negative and the patient was treated with IV vancomycin. mNGS detected several pathogens, with *Klebsiella* spp. showing the highest titer at 5.8 GPM. Three weeks prior to the relevant admission, the patient had severe sepsis requiring treatment in the intensive care unit, following the ureteral stone removal. Notably, *Klebsiella pneumoniae* had been cultured from both blood and urine during this admission, suggesting a likely relevance of the mNGS result.

The third patient (p072), a 64-year-old male, was admitted upon suspicion of pyelonephritis, but blood and urine cultures taken at admission were negative, likely due to the patient already receiving pivmecillinam ordered from his general practitioner. At admission, the treatment was escalated to IV ampicillin with good clinical response. mNGS was positive for *E. coli* (2.8 GPM).

### Potential clinical impact

The mNGS results for the 22 patients, where the result was either confirmed by blood culture or deemed relevant by a clinical microbiologist, were analyzed for potential clinical impact. In a real-time setting, the mNGS result could have affected the antimicrobial treatment in 13 cases (59%) subgrouping to five changes from ineffective to effective treatment, seven escalations from oral to IV, and one addition of an extra antimicrobial (more details in Table S3). The five cases where a change from ineffective to effective treatment could have been facilitated are particularly interesting. In patients from BSI-confirmed, this was achieved by identifying an unsuspected pathogen more rapidly than the blood culture (*n* = 4), while it in patients from BSI-suspected was achieved by identifying an unsuspected pathogen not detected by blood culture (*n* = 1). These findings should be interpreted with great caution due to the small cohort size, batched sample analysis, and observational study setup.

## DISCUSSION

In recent years, multiple studies have assessed the use of cfDNA mNGS for BSI diagnostics of patients admitted to e.g., the intensive care unit (14, 25), the emergency ward (13), hematology (21), and pediatric departments (26). In this study, we showed that rapid Nanopore mNGS could be a feasible supplement to blood culturing, as the test confirmed all positive blood cultures (*n* = 11) and provided a relevant pathogen identification in an additional 11 patients. In general, for patients admitted to the emergency ward, no or few microbiological test results (e.g., from primary care or previous admissions) are available to guide empirical antimicrobial therapy, and rapid and sensitive infection diagnostics are most welcomed by treating physicians for guidance of effective antimicrobial therapy and to aid antimicrobial stewardship initiatives (27–29).

The mNGS results reported in this study constitute both common and rare pathogens from various suspected body site origins including UTIs, LRTIs, and intra-abdominal abscesses, demonstrating applicability across a broad range of clinical syndromes. To the best of our knowledge, only a single study with four patients has evaluated the use of Nanopore sequencing for mNGS BSI diagnostics directly from blood plasma (30), and our study provides additional evidence for the feasibility of using the platform to reduce turnaround time by including patients with negative blood cultures. We also introduce a quantitative measure of microbial cfDNA in the original sample as a way of standardizing results between patients (and possibly cohorts) and to allow for an improved clinical interpretation of results.

Validation and interpretation of mNGS results in patients with a negative blood culture is difficult, and the many additional findings have led to questions on the clinical relevance of these identifications (31, 32). In the BSI-suspected group from this study, the high number of microbiological tests from other sites that confirm the mNGS result and the fact that patients were included in this group upon suspicion of a systemic infection support the relevance of mNGS pathogen identifications. Furthermore, no microbes were identified with mNGS in patients from the BSI-absent group. In addition to the finding of common pathogens, mNGS also identified microbes that were unlikely to be responsible for the acute BSI. This includes *Helicobacter pylori* and *C. difficile*, and such findings necessitate that results are interpreted with caution.

We also hypothesize that a fast and sensitive mNGS test has the potential to impact a large fraction of antimicrobial treatments, albeit these findings should be interpreted with caution given the observational study setup and small cohort size. While mNGS tests for clinical microbiology diagnostics are expensive to conduct (33), the added value of increased sensitivity and fast turnaround time on the antimicrobial treatment may reduce mortality and length of stay to justify the cost in selected patient groups. However, larger studies with intervention are required to answer these questions. We see this test best applied to vulnerable patient groups where etiology determination is crucial for patient survival.

This study has several limitations. First, only patients who could provide informed consent were included, and the patients who were in septic shock were thus excluded. Second, the study was conducted in a non-real-time setup with batching of samples for analysis after all patients were included in the cohort. Third, only a subset of the patients included were analyzed, selecting the patients with the highest likelihood of a true BSI, and the performance may be different for the BSI-unlikely group. Fourth, the study only analyzed four patients with no suspicion of a BSI (BSI-absent), and a comprehensive evaluation of specificity should include more patients to this group. Fifth, for several patients, the mNGS assay detected multiple pathogens, which may require additional interpretation by a clinical microbiologist. Finally, the setup of the study did not allow us to conduct follow-up microbiological tests to investigate mNGS findings when discordant with results from blood culturing.

We demonstrated that Nanopore mNGS can identify pathogens clinically relevant to the acute infection in twice as many patients as gold-standard blood culture and that the method detects a broad range of etiologies. In summary, this study provides a proof-of-concept for the use of Nanopore cfDNA metagenomics as a diagnostic tool for patients with a BSI, and the study encourages the method to be evaluated in larger cohorts and subjected to comprehensive analytical validation.

## Data Availability

Individual patient metadata is shared in Tables S1 and S3. Microbial DNA sequencing data from positive mNGS tests is deposited in NCBI SRA and is accessible with BioProject identifier PRJNA1108520. The study protocol is available at https://vbn.aau.dk/da/datasets/study-protocol-for-a-new-method-using-rapid-nanopore-metagenomic-.

## SUPPLEMENTAL MATERIAL

Supplemental material spectrum.03295-24-s0001.docx Fig. S1; Tables S1 to S3.

ASM does not own the copyrights to Supplemental Material that may be linked to, or accessed through, an article. The authors have granted ASM a non-exclusive, world-wide license to publish the Supplemental Material files. Please contact the corresponding author directly for reuse.
