# Supplementary material for: Application of rapid Nanopore metagenomic cell-free DNA sequencing to diagnose bloodstream infections: a prospective observational study
Source: Microbiol Spectr. 2025 Mar 26;13(5):e03295-24. doi: 10.1128/spectrum.03295-24 (PMC12054037; doi:10.1128/spectrum.03295-24)
Supplement: Supplemental material — Fig. S1; Tables S1 to S3. [file spectrum.03295-24-s0001.docx]

**Supplementary materials**

**Application of rapid Nanopore metagenomic cell-free DNA sequencing to diagnose bloodstream infections: a prospective observational study**

**Nielsen et al.**

Table of contents

[Supplementary Figures 3](#_Toc183435864)

[Figure S1. Clinical characteristics of patients who were analyzed and excluded. 3](#_Toc183435865)

[Supplementary Tables 4](#_Toc183435866)

[Table S1. Detailed metadata on clinical presentation and antibiotics for all patients. 4](#_Toc183435867)

[Table S2. Read depth and plasma cfDNA concentration for all patients and blood donors. 7](#_Toc183435868)

[Table S3. Detailed metadata on the assessment of relevance of mNGS findings. 9](#_Toc183435869)

[References 11](#_Toc183435870)

# Supplementary Figures

**
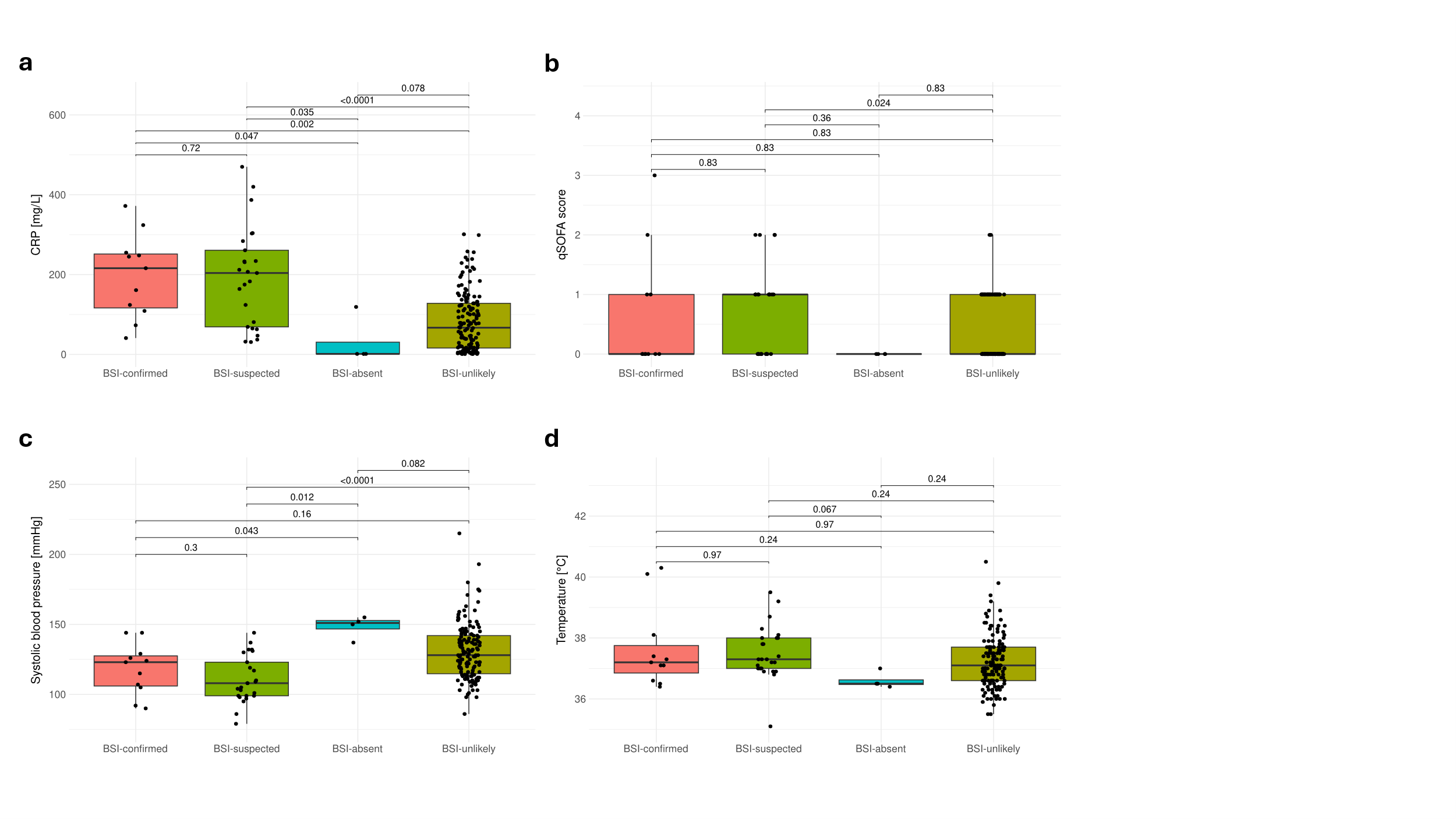
**

## Figure S1. Clinical characteristics of patients who were analyzed and excluded.

a) CRP at blood culture for BSI-confirmed, BSI-suspected, BSI-absent, and BSI-unlikely groups. b) qSOFA score at blood culture for the four groups. c) Systolic blood pressure at blood culture for the four groups. d) Temperature at blood culture for the four groups.

# Supplementary Tables

## Table S1. Detailed metadata on clinical presentation and antibiotics for all patients.

| Patient | Suspected primary infection site | CRP (mg/L) | Systolic blood pressure  (mmHg) | Temperature (°C) | WBC (10³ cells/mm³) | RF (1/min) | GCS | qSOFA | Antibiotics prior to admission | Antibiotics during admission |
| --- | --- | --- | --- | --- | --- | --- | --- | --- | --- | --- |
| p001 | LRTI | 109 | 124 | 40.1 | 10.3 | 30 | 13 | 2 |  | Gentamicin \| Cefuroxime \| Benzylpenicillin \| Ampicillin \| Piperacillin/tazobactam |
| p002 | UTI | 216 | 90 | 36.6 | 10.7 | 24 | 14 | 3 |  | Gentamicin \| Piperacillin-tazobactam \| Meropenem \| Ciprofloxacin \| Ampicillin |
| p020 | UTI | 161 | 115 | 36.4 | 14.8 | 20 | 15 | 0 |  | Piperacillin-tazobactam \| Mecillinam \| Pivmecillinam |
| p028 | LRTI | 255 | 105 | 37.1 | 24.8 | 24 | 15 | 1 |  | Amoxicillin \| Piperacillin/tazobactam |
| p098 | Idiopathic pancreatitis | 73 | 144 | 37.3 | 14.5 | 16 | 15 | 0 |  | Piperacillin-tazobactam \| Metronidazole |
| p104 | Infection with unknown focus | 41 | 123 | 36.5 | 7.5 | 16 | 15 | 0 |  | Piperacillin-tazobactam \| Ciprofloxacin \| Vancomycin |
| p139 | Infection with unknown focus | 248 | 129 | 37.4 | 5.3 | 18 | 15 | 0 |  | Piperacillin-tazobactam \| Ciprofloxacin |
| p141 | Knee | 324 | 144 | 37.2 | 5.3 | 12 | 15 | 0 |  | Cloxacillin \| Dicloxacillin \| Benzylpenicillin \| Cefuroxime |
| p172 | UTI | 245 | 107 | 40.3 | 17.1 | 16 | 15 | 0 |  | Gentamicin \| Ampicillin \| Piperacillin-tazobactam \| Pivmecillinam \| Phenoxymethylpenicillin |
| p175 | Infection with unknown focus | 124 | 92 | 38.1 | 13.6 | 19 | 15 | 1 |  | Piperacillin-tazobactam \| Vancomycin |
| p183 | UTI | 372 | 126 | 37.1 | 19.4 | 18 | 15 | 0 |  | Piperacillin-tazobactam \| Ampicllin \| Metronidazole |
| p012 | Infection with unknown focus | 204 | 86 | 36.8 | 7.6 | 23 | 15 | 2 | Metronidazole | Nystatin \| Amoxicillin \| Piperacillin/tazobactam |
| p018 | PICC-line infection | 37 | 95 | 36.9 | 4.8 | 12 | 15 | 1 |  | Benzylpenicillin \| Fluconazole \| Ampicillin \| Anidulafungin |
| p019 | LRTI | 284 | 103 | 38 | 10.8 | 22 | 15 | 1 |  | Ampicillin \| Gentamicin \| Piperacillin/tazobactam |
| p022 | Infection with unknown focus | 175 | 101 | 35.1 | 18.1 | 16 | 15 | 0 | Phenoxymethyl-penicillin | Ampicillin \| Piperacillin-tazobactam \| Moxifloxacin |
| p027 | Skin | 63 | 99 | 37.3 | 10.9 | 18 | 15 | 1 |  | Fluconazole \| Clarithromycin \| Piperacillin-tazobactam \| Dicloxacillin |
| p047 | LRTI | 124 | 130 | 39.2 | 23.3 | 22 | 15 | 1 |  | Benzylpenicillin \| Piperacillin-tazobactam\| Amoxicillin |
| p049 | LRTI | 420 | 131 | 37.3 | 27.7 | 16 | 15 | 0 |  | Cefuroxime \| Clarithromycin \| Roxithromycin \| Moxifloxacin |
| p072 | UTI | 207 | 144 | 37 | 15.5 | 16 | 15 | 0 | Pivmecillinam | Ampicillin \| Pivampicillin \| Pivmecillinam \| Gentamicin |
| p091 | UTI | 261 | 108 | 37.2 | 12.3 | 14 | 15 | 0 |  | Piperacillin-tazobactam \| Pivmecillinam |
| p092 | UTI | 212 | 108 | 37 | 10 | 14 | 15 | 0 |  | Piperacillin-tazobactam \| Pivmecillinam |
| p105 | LRTI | 233 | 137 | 38.7 | 23.4 | 22 | 15 | 1 | Azithromycin | Clarithromycin \| Benzylpenicillin \| Piperacillin-tazobactam \| Amoxicillin \| Moxifloxacin |
| p106 | LRTI | 387 | 117 | 37 | 13.1 | 22 | 15 | 1 | Unknown antibiotic | Piperacillin-tazobactam \| Phenoxymethylpenicillin |
| p114 | LRTI | 69 | 97 | 37.2 | 12.3 | 22 | 15 | 2 |  | Benzylpenicillin \| Amoxicillin |
| p127 | Diverticulitis | 183 | 99 | 37.1 | 16.3 | 22 | 15 | 2 |  | Metronidazole \| Benzylpenicillin \| Gentamicin \| Amoxicillin |
| p128 | LRTI | 470 | 99 | 36.9 | 12.2 | 20 | 15 | 1 |  | Benzylpenicillin \| Phenoxymethylpenicillin |
| p136 | Psoas | 303 | 105 | 37.8 | 15.4 | 12 | 15 | 0 | Unknown antibiotic | Metronidazole \| Piperacillin-tazobactam\| Fluconazole |
| p140 | UTI | 81 | 132 | 38 | 13.6 | 16 | 15 | 0 | Pivmecillinam | Ampicillin \| Gentamicin \| Pivmecillinam |
| p143 | Decubitus os sacrum | 231 | 110 | 37.8 | 14.1 | 18 | 14 | 1 |  | Cefuroxime \| Amoxicillin |
| p146 | Skin | 65 | 104 | 36.9 | 10.5 | 18 | 15 | 0 |  | Benzylpenicillin \| Cloxacillin \| Phenoxymethylpenicillin |
| p150 | LRTI | 31 | 109 | 39.5 | 1.4 | 18 | 15 | 0 |  | Ciprofloxacin \| Piperacillin-tazobactam \| Gentamicin \| Meropenem |
| p151 | Infection with unknown focus | 304 | 123 | 38.1 | 13 | 16 | 15 | 0 |  | Cefuroxime \| Trimethoprim |
| p156 | LRTI | 47 | 79 | 37.4 | 28.9 | 20 | 15 | 1 |  | Piperacillin-tazobactam \| Amoxicillin |
| p162 | LRTI | 32 | 98 | 37.3 | 9.4 | 28 | 15 | 2 |  | Meropenem \| Ciprofloxacin |
| p164 | UTI | 234 | 119 | 38.3 | 7.3 | 14 | 15 | 0 |  | Piperacillin-tazobactam \| Ciprofloxacin |
| p173 | UTI | 164 | 132 | 38 | 10 | 14 | 15 | 0 |  | Vancomycin \| Pivmecillinam \| Ciprofloxacin |
| p016 |  | 119 | 152 | 36.5 | 9.8 | 14 | 15 | 0 |  |  |
| p059 |  | 1.2 | 155 | 36.4 | 6.4 | 20 | 15 | 0 |  |  |
| p068 |  | 1.3 | 150 | 36.5 | 10.1 | 16 | 15 | 0 |  |  |
| p120 |  | 1.3 | 137 | 37 | 9.6 | 16 | 15 | 0 |  | Dicloxacillin |

Abbreviations: RF = Respiratory frequency, WBC = White blood cell count, CRP = C-reactive protein, qSOFA = Quick Sequential Organ Failure Assessment (1), GCS = Glasgow Coma Scale.

## Table S2. Read depth and plasma cfDNA concentration for all patients and blood donors.

| Patient | DNA concentration (ng/mL) | Read depth (M) | Average read quality (Q-score) | Read length N50 |
| --- | --- | --- | --- | --- |
| p001 | 9.6 | 2.69 | 18.9 | 167 |
| p002 | 3.6 | 2.10 | 18.8 | 172 |
| p012 | 14 | 14.2 | 19.0 | 181 |
| p016 | 3.8 | 5.72 | 18.7 | 172 |
| p018 | 9.0 | 5.22 | 19.0 | 171 |
| p019 | 71 | 10.2 | 19.1 | 210 |
| p020 | 15 | 1.15 | 18.7 | 165 |
| p022 | 4.1 | 8.11 | 18.0 | 171 |
| p027 | 13 | 18.7 | 19.1 | 173 |
| p028 | 27 | 9.15 | 18.6 | 167 |
| p047 | 25 | 21.4 | 18.6 | 167 |
| p049 | 19 | 5.94 | 18.5 | 175 |
| p059 | 3.6 | 9.94 | 18.7 | 169 |
| p068 | 2.7 | 0.629 | 18.5 | 173 |
| p072 | 19 | 7.18 | 19.2 | 182 |
| p091 | 8.4 | 10.3 | 19.0 | 168 |
| p092 | 8.5 | 5.44 | 18.8 | 177 |
| p098 | 7.4 | 9.99 | 18.9 | 181 |
| p104 | 2.4 | 16.9 | 18.8 | 166 |
| p105 | 22 | 5.61 | 18.7 | 192 |
| p106 | 27 | 6.48 | 19.0 | 170 |
| p114 | 6.0 | 18.2 | 18.7 | 172 |
| p120 | 0.60 | 1.67 | 16.7 | 168 |
| p127 | 2.8 | 11.8 | 18.4 | 168 |
| p128 | 35 | 17.3 | 18.7 | 180 |
| p136 | 17 | 18.3 | 19.3 | 177 |
| p139 | 10 | 9.40 | 19.3 | 181 |
| p140 | 10 | 16.6 | 19.4 | 174 |
| p141 | 35 | 5.65 | 18.6 | 212 |
| p143 | 19 | 11.3 | 18.9 | 174 |
| p146 | 2.3 | 11.7 | 19.6 | 173 |
| p150 | 150 | 6.30 | 19.9 | 207 |
| p151 | 16 | 11.6 | 20.1 | 174 |
| p156 | 11 | 4.22 | 20.0 | 172 |
| p162 | 8.3 | 12.7 | 19.8 | 171 |
| p164 | 22 | 12.6 | 19.9 | 170 |
| p172 | 13 | 15.5 | 19.9 | 171 |
| p173 | 14 | 10.4 | 20.0 | 172 |
| p175 | 9.1 | 21.0 | 20.0 | 172 |
| p183 | 140 | 10.5 | 20.0 | 175 |
| d001 | 4.3 | 6.47 | 18.3 | 172 |
| d002 | 4.5 | 10.3 | 18.6 | 173 |
| d003 | 2.1 | 8.57 | 18.2 | 170 |
| d004 | 1.3 | 10.4 | 19.4 | 173 |
| d005 | 1.1 | 5.48 | 18.2 | 190 |
| d006 | 2.4 | 13.6 | 18.7 | 173 |
| d007 | 1.4 | 3.24 | 18.3 | 170 |
| d008 | 0.8 | 14.5 | 18.2 | 164 |
| d009 | 7.5 | 11.6 | 19.3 | 173 |
| d010 | 2.3 | 16.7 | 19.4 | 172 |
| d011 | 4.2 | 17.6 | 20.0 | 170 |
| d012 | 2.7 | 2.00 | 18.9 | 170 |

## Table S3. Detailed metadata on the assessment of relevance of mNGS findings.

| Patient | Blood culture result | Secondary microbiology result | mNGS result (GPM) | Clinical relevance of mNGS result | Potential impact on antibiotic treatment | Comments related to treatment impact |
| --- | --- | --- | --- | --- | --- | --- |
| p001 | *Enterococcus faecalis* | Urine: *E. faecalis* | *E. faecalis* (1) | Confirmed | Change | From gentamicin and cefuroxime to ampicillin |
| p002 | *Escherichia coli* | Urine: *E. coli*, Urine: *Enterococcus* spp. | *E. coli* (20.8) | Confirmed | None |  |
| p020 | *E. coli* | Urine: *E. coli* | *E. coli* (1.6) | Confirmed | None |  |
| p028 | *E. coli* | Urine: *E. coli* | *E. coli* (41.3) | Confirmed | None |  |
| p098 | *E. coli* |  | *E. coli* (0.9) | Confirmed | None |  |
| p104 | *Staphylococcus epidermidis* |  | *S. epidermidis* (52.4)  KSHV (1) | Confirmed | Change | From piperacillin-tazobactam to vancomycin |
| p139 | *E. coli* |  | *E. coli* (102.2)  *Enterococcus faecium* (0.7)  CMV (26.6) | Confirmed | Addition | Addition of antiviral treatment |
| p141 | *Staphylococcus aureus* | Urine: *S. aureus*, Joint: *S. aureus* | *S. aureus* (31) | Confirmed | Change | Patient was discharged without any antibiotic treatment and readmitted and started cloxacillin the next day when the blood culture was positive. |
| p172 | *E. coli* | Urine: *E. coli* | *E. coli* (59.4) | Confirmed | None |  |
| p175 | *S. epidermidis* | Urine: *Enterococcus spp.* | *S. epidermidis* (53.8) | Confirmed | Change | From piperacillin-tazobactam to vancomycin |
| p183 | *E. coli* | Urine: *E. coli,* Feces: *Clostridiodes difficile* | *E. coli* (320.8)  *Citrobacter koseri* (2.4) | Confirmed | None |  |
| p019 |  |  | *E. coli* (58.7)  *Haemophilus influenzae* (13.4)  *Rothia dentocariosa* (4.5)  *Streptococcus anginosus* (4.6)  *Veillonella atypica* (6.5) | Probable | None |  |
| p049 |  |  | *Proteus mirabilis* (0.5) | Possibleᵃ | None |  |
| p072 |  |  | *E. coli* (2.8) | Probable | Escalation | Extended duration of IV antibiotic treatment |
| p091 |  | Urine: *E. coli* | *E. coli* (1.8) | Probable | Escalation | Extended duration of IV antibiotic treatment |
| p092 |  | Urine: *E. coli* | *E. coli* (0.9)  *H. pylori* (13.8) | Probable | None |  |
| p105 |  | BAL: *Pseudomonas aeruginosa* | *P. aeruginosa* (121.6) | Probable | Change | From penicillin and clarithromycin to piperacillin-tazobactam and ciprofloxacin |
| p127 |  |  | *Raoultella planticola* (0.6) | Probable | Escalation | Peroral escalation from amoxicillin to ciprofloxacin |
| p128 |  |  | *S. pneumoniae* (3.5) | Probable | Escalation | Extended duration of IV antibiotic treatment |
| p140 |  | Urine: *E. coli* | *E. coli* (5.9) | Probable | Escalation | Extended duration of IV antibiotic treatment |
| p143 |  | Urine: *Enterococcus spp.,* Wound: *Proteus spp.* | *C. difficile* (0.5) | Unlikely | None |  |
| p164 |  | Urine: *E. coli* | *E. coli* (8) | Probable | Escalation | Extended duration of IV antibiotic treatment |
| p173 |  | Urine: *Enterococcus spp.* | *Klebsiella spp.* (5.8)  *Acinetobacter bereziniae* (1)  *Brevibacterium paucivorans* (2)  *Leuconostoc pseudomesenteroides* (1.1)  *Pantoea dispersa* (1.1)  *Serratia spp.* (3.1) | Probable | Escalation | Extended duration of IV antibiotic treatment |

Abbreviations: GPM = Genome Equivalents per microliter, KSHV = Kaposi's sarcoma-associated herpesvirus, CMV = Cytomegalovirus, BAL = bronchoalveolar lavage.

ᵃConsistent with a bronchial lavage culture from a previous hospital admission, which supports the finding of *P. mirabilis* in a patient with LRTI focus.

# References

1. Lambden S, Laterre PF, Levy MM, Francois B. 2019. The SOFA score-development, utility and challenges of accurate assessment in clinical trials. Crit Care 23:374.
